# The tomato seed microbiome is mainly shaped by host genotype and production site

**DOI:** 10.1128/msystems.01441-25

**Published:** 2025-12-18

**Authors:** Xiaoyulong Chen, Expedito Olimi, Carolina Lobato, Gabriele Berg, Tomislav Cernava

**Affiliations:** 1 College of Life Sciences, Guizhou University 71206, Guiyang, China; 2 Institute of Environmental Biotechnology, Graz University of Technology 27253, Graz, Austria; 3 School of Biological Sciences, Faculty of Environmental and Life Sciences, University of Southampton 7423, Southampton, United Kingdom; 4 Leibniz Institute for Agricultural Engineering and Bioeconomy (ATB) 28398, Potsdam, Germany; 5 Institute for Biochemistry and Biology, University of Potsdam 26583, Potsdam, Germany; Boise State University, Boise, Idaho, USA

**Keywords:** plant microbiome, *Solanum lycopersicum*, plant endophytes, seed endophytes, genotype variation

## Abstract

The seed microbiome plays a crucial role in plant health and productivity, yet the extent to which the plant genotype influences its composition remains unclear. We conducted a large-scale study using 100 tomato (*Solanum lycopersicum* L.) genotypes from 12 geographical locations in China, subjecting all seeds to the same processing to assess seed microbiome plasticity. The plant genotype was identified as the primary factor shaping microbiome structure (*R*² = 0.56, *P* = 0.001), followed by geographic location (*R*² = 0.11, *P* = 0.001), and insect resistance of the cultivar (*R*² = 0.07, *P* = 0.001). A rather small core microbiome of 21 amplicon sequence variants (ASVs) was shared across all tomato genotypes. Ubiquitous seed microbiome members found in 90% of the samples included *Pseudomonas*, *Lactobacillus*, *Leuconostoc*, and *Ralstonia*. A Random Forest modeling approach showed that tomato traits and their production environment can be predicted via seed microbiome features; core microbiome members, including *Lactobacillus* and *Pseudomonas,* were connected to specific tomato traits. This study unveils key factors influencing seed microbiome assembly and emphasizes the crucial role of host traits that can enable new venues for seed microbiomes in plant breeding and sustainable crop production.

**IMPORTANCE:**

Seeds not only carry the plant's genetic material but also host distinct microbial communities that can influence early plant growth and performance. In a large-scale study involving 100 tomato genotypes collected from 12 geographical locations in China, we examine how plant genotype shapes the seed microbiome. The research findings reveal that plant genotype, more than location or parents' geography, primarily influences microbial community structure (*R*² = 0.56 vs 0.11). These findings highlight the strong association between host genetics and seed microbiome assembly. Understanding these interactions provides valuable opportunities for integrating microbiome-based strategies into plant breeding and crop improvement programs, ultimately supporting more resilient and sustainable agricultural systems.

## INTRODUCTION

Plants host highly diverse and phylogenetically structured microbial communities—the plant microbiota. The microbiota is important in ensuring plant health, fitness, resilience, and productivity (1, 2); specific members of the microbiota can complement their host’s functioning and alleviate biotic (3, 4), as well as abiotic stress (5, 6). Consequently, plants evolved mechanisms to recruit and retain particular microbiota components that are crucial for their survival (1, 7). There is an urgent need to reduce the use of synthetic chemicals in agriculture, which drives a growing interest in incorporating microbe-based solutions in agricultural systems (2). Harnessing plant microbiota functions is not only seen as a potential substitute for conventional crop production strategies, which heavily rely on synthetic agrochemical inputs, but also to ensure food security in the future (8, 9) and facilitate crop resilience toward the effects of climate change (6, 10). The delivery of microbial solutions in agricultural systems will likely make increasingly use of seeds as vessels for various bioproducts (11, 12). Thus, studying seed microbiomes is opening new research frontiers guided by state-of-the-art data science for improving microbiome-guided crop breeding and selection (13, 14).

Seeds are sexually derived structures of spermatophytes that mark the end and beginning of the plant cycle (15). Besides their role as a conduit for propagating plant genetic information, seeds are reservoirs of diverse and highly specialized microbial communities, integral to plant success, and can vary between plant hosts (16–18). The occurrence of seed microbes is compartmentalized within seed tissues (endophytes) and on seed surfaces (epiphytes) (15, 19, 20). Endophytes constitute a major portion of the vertically transferred seed microbiome (15, 21), which may create a lasting impact across plant generations (22, 23); for some plant families, the heritability of the assembled seed microbiome fluctuates across plant generations (24). Epiphytes, on the other hand, are mainly transferred horizontally and may or may not become internalized within seed tissues for vertical transmission (19). The transferred seed microbiota plays a crucial role in providing the first starter inoculum required for microbiota assembly (20, 25). Current empirical evidence shows that the seed microbiota, and particularly endophytes, are mainly constituted of plant-beneficial members (26) that contribute to host tolerance of a/biotic stress, plant vigor, and productivity (13, 27). For instance, the seed endophytic bacterium *Sphingomonas melonis* confers holistic protection against infection by *Burkholderia plantarii* in rice, being referred to as a “soterobiont” (28).

Tomato, a crop belonging to the *Solanaceae* plant family, is phylogenetically diverse and geographically widespread, owing to its nutritional and economic importance. Yet, the crop is threatened by a plethora of a/biotic challenges, some of which can be addressed using microbiome-based solutions. Moreover, recent studies have indicated that tomato has one of the most diverse seed microbiomes (16). This makes it suitable to investigate hypotheses in the realm of plant-microbe interactions (29). In this large-scale study with 100 tomato genotypes and extensive metadata, we examine the plasticity in the tomato seed microbiome for plants with differing genetic backgrounds and grown in 12 geographic locations in China. We used predictive models to explore microbial markers associated with various host traits, including insect and disease resistance, tomato yield, 1,000 seed weight in grams, number of ovaries, as well as berry color, shape, and taste. The overarching goal was to provide concrete insights into the influence of host traits and environment in shaping the seed microbiome by leveraging the metadata-extensive tomato seed microbiome data set. The research presents an opportunity to explore the tomato seed microbiome for beneficial members and provides the basis for using seeds as a conduit for delivering microbiome-based solutions.

## MATERIALS AND METHODS

### Seed material, experimental design, sample collection, and processing

**Fig 1:**
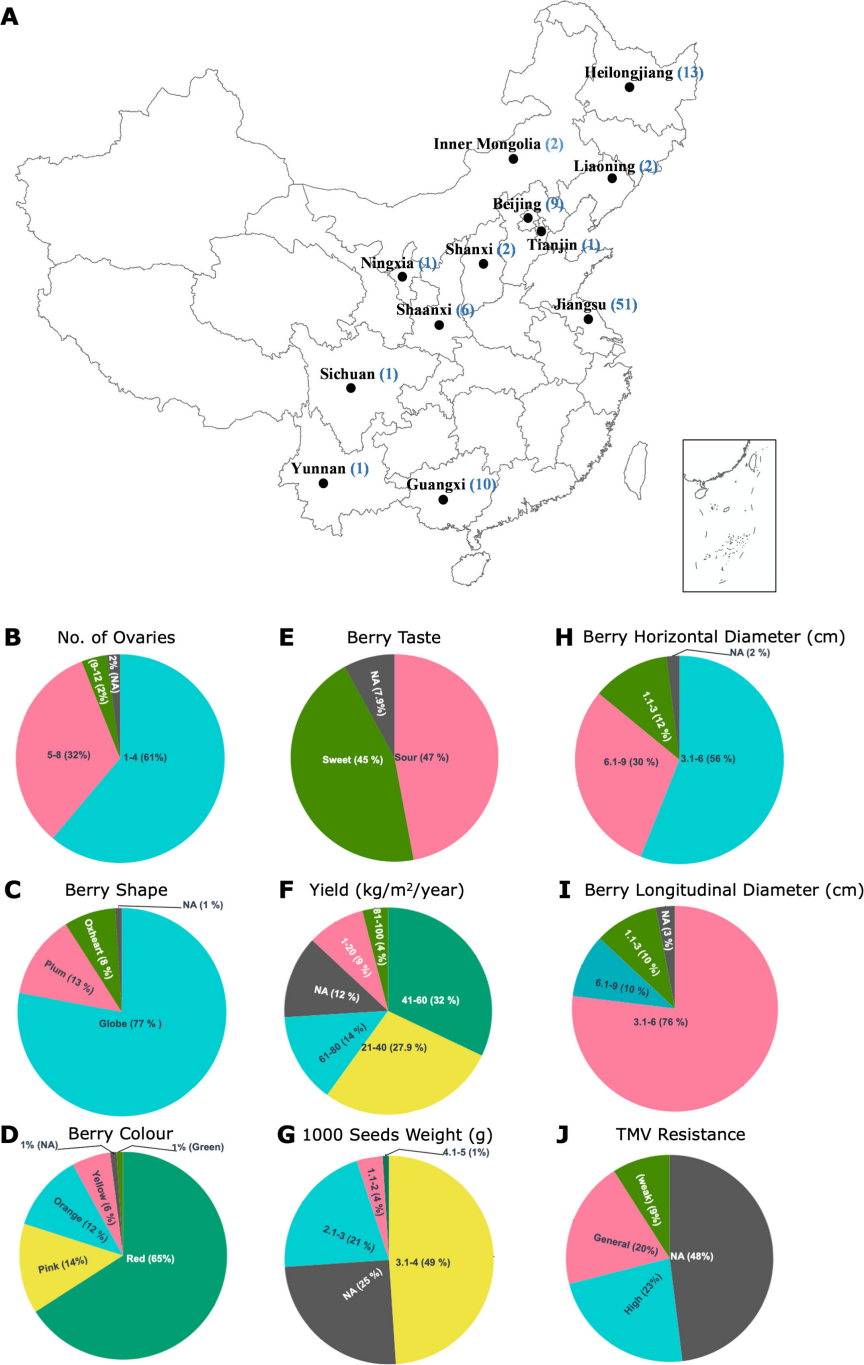
Overview of the geographic origins of the tomato seeds and the accompanying metadata. The different locations of seed production (*n* = 12) are visualized on a map of China (**A**) together with the number of genotypes (brackets) obtained from each location. The number of ovaries (**B**), berry shape (**C**), color (**D**), and taste (**E**); as well as yield (**F**), 1,000 seed weight (**G**), and interval groups of the horizontal (**H**), and longitudinal (**I**) berry diameter were captured in the available metadata. Additional metadata was available for disease resistance, such as resistance to TMV (**J**). The map was drawn using the PHYLOGEO package (30), utilizing the phyloseq-formatted data set in R Studio.

This study presents, so far, the largest tomato seed microbiome dataset, focusing on examining the bacterial community of 100 genotypes, grown in 12 Chinese provinces (Fig. 1A). Briefly, tomato seed samples were obtained from seed banks at various agricultural research institutes in China, including Jiangsu (*n* = 51), Guangxi (*n* = 10), Beijing (*n* = 10), Heilongjiang (*n* = 13), Inner Mongolia (*n* = 2), Sichuan (*n* = 1), Liaoning (*n* = 2), Ningxia (*n* = 1), Shanxi (*n* = 2), Tianjin (*n* = 1), Shaanxi (*n* = 6), and Yunnan (*n* = 1). The seeds were extracted from several fully ripe tomato fruits, which were obtained from batches of several plants for better representativeness. Seeds were washed with sterile water and left to dry in a dry chamber (15°C, RH ≤ 45%). Additional experimental metadata included the number of ovaries, berry shape, color, berry taste, yield (kg/m^2^/year), 1,000 seed weight (g), berry horizontal and longitudinal diameter (cm), and resistance against tomato mosaic virus (TMV), as shown in Fig. 1B through J. The tomato resistance status against insect pests, such as aphids, whiteflies, and bollworms, is recorded in the sample metadata as general aphid-, whitefly-, and bollworm-resistance, as well as general resistance to other insects. An elaborate description of experimental metadata is presented in Data S1 at https://doi.org/10.6084/m9.figshare.30823943 . Sample preparation employed aseptic techniques during seed processing for microbial DNA extraction from the seed endosphere. Briefly, 0.3–0.5 g of seed samples (*n* = 12 per genotype) were surface sterilized by washing with 4% NaOCl for 5 min, followed by rinsing them three times with sterile distilled water. The last wash was plated on nutrient agar to confirm sterility. For sample processing, seeds were homogenized using a mortar and pestle. Total microbial DNA was extracted using the FastDNA Spin Kit for Soil (MO BIO Laboratories, USA), following the manufacturer’s instructions, followed by quantification using a NanoDrop 2000c spectrophotometer (Thermo Scientific, Waltham, MA, USA) and storage at −20°C. Total community DNA was extracted from 12 biological replicates for each tomato genotype.

### Seed bacterial community analysis by 16S rRNA gene fragment profiling

High-throughput amplicon sequencing of a bacterial taxonomic marker (16S rRNA gene) was employed to profile the seed microbiome (bacterial community). Here, universal primers were used in combination with PNA PCR blockers (pPNA: 5′-GGCTCAACCCTGGACAG-3′ and mPNA: 5′-GGCAAGTGTTCTTCGGA-3′) to reduce co-amplification of plastid DNA (31). The hypervariable V4 location of the 16S rRNA gene was amplified with the primer pair 515F/806R (32), carrying sample-specific tags. The reaction mixture for the PCR (30 µL) contained 1× Taq &GO (MP Biomedicals, Solon, OH, USA), 0.2 µM of each primer, 0.75 µM of pPNA and mPNA (PNA Bio, Thousand Oaks, CA, USA), respectively, and 2 µL of template DNA (96°C, 5 min; 30 cycles of 96°C, 1 min; 78°C, 5 s for PNA annealing; 54°C, 1 min; 74°C, 1 min; and elongation at 74°C, 10 min). PCR products of three independent reactions were pooled in equal volumes and purified by employing the Wizard SV Gel and PCR Clean-Up System (Promega, Madison, WI, USA). Negative controls without template DNA were included and did not result in amplification; they were therefore not included in the sequencing pool. Amplicon libraries were sequenced with a paired-end approach on an Illumina PE250 platform (2 × 250 bp) by the sequencing provider Novogene (Beijing, China), using a sequencing depth of 100,000 quality-filtered (Q30 ≥ 75%) reads per sample.

Raw amplicon sequencing data were denoised, joined, delineated into amplicon sequence variants (ASVs), and assigned taxonomy in the QIIME 2 (v2023.9.1) environment (https://qiime2.org) (33). Paired-end reads were demultiplexed and primers removed using cutadapt (34). Demultiplexed sequence reads were quality checked using fastQC, followed by MultiQC-based summarization (35). The data sets were then quality filtered, trimmed, denoised, merged, and chimeras removed using the DADA2 v1.26.0 pipeline (36), resulting in ASVs and a table of feature counts. The quality filtering was performed based on phred33 scores, and reads were trimmed and truncated using parameters (--p-trim-left-f 20 --p-trim-left-r 20 --p-trunc-len-f 180 --p-trunc-len-r 180). Representative sequences were subsequently taxonomically classified by alignment against the SILVA132 reference database (37, 38), using the VSEARCH algorithm (39), implemented using the q2-feature-classifier command (40). The SILVA132 database was composed of pre-formatted taxonomic data using the RESCRIPT package (https://github.com/bokulich-lab/RESCRIPt) (41). In addition, sequence data sets were used to generate a rooted phylogenetic tree using the plugin (QIIME PHYLOGENY ALIGN-TO-TREE-MAFFT-FASTTREE) with default parameters.

### Statistical analysis and modeling

Downstream analyses were performed using R v4.2.2 (http://www.r-project.org) in RStudio v1.1.423, using the packages PHYLOSEQ, VEGAN, TIDYVERSE, GGPLOT2, etc., as specified in the publicly available R script (https://github.com/kaboyo/Tomato-seed-microbiome). The feature table and taxonomic information, as well as experimental metadata and phylogenetic tree, were analyzed using PHYLOSEQ v1.46.0 (42) and VEGAN v2.5.7 (43) packages. Filtering to exclude non-target taxa, like unassigned sequences, chloroplasts, and mitochondria, was performed to only retain the bacterial portion of the seed microbiome. To reduce community inflation caused by rare or low-abundance ASVs, further filtering was applied using a prevalence threshold of 0.25% within all samples, as previously described (44). Overall, amplicon sequencing resulted in 27,281,011 reads, corresponding to 15,421 bacterial ASVs. Normalization by rarefying data sets was performed using 1,000 reads per sample as the minimum sampling depth for the estimation of bacterial alpha diversity across the samples (see Fig. S1A at https://doi.org/10.6084/m9.figshare.30823943). For beta diversity, various normalization approaches, including total sum scaling (TSS), cumulative sum scaling (CSS), as well as Hellinger transformation, were performed.

Alpha diversity analysis involved computing various indices, like richness (observed ASVs), Shannon index, evenness, and Simpson index. These indices estimate community diversity and richness. Additionally, effective diversity estimates were also computed (45). Estimating the effective diversity provides a more nuanced measure of community diversity than standalone indices, like species richness, Shannon, or Simpson indices. All alpha diversity indices (including “effective diversity”) were computed using the procedure and adapted R script that is described in the RHEA package (46). Moreover, correlation analysis of seed bacterial diversity and richness with sequencing depth was also conducted to estimate the minimum number of sequences necessary to achieve the desired diversity coverage for the seed microbiome experiments. Non-parametric (rank-based) Kruskal-Wallis test was used for the assessment of statistical differences in alpha diversity indices between the various experimental variables. Dunn’s post hoc test, coupled with Bonferroni’s correction of *P* values, was performed for factors that showed global significant differences (*P* < 0.05). The two tests were performed using the KRUSKAL TEST() and DUNN TEST() functions in RSTATIX (47).

Bacterial community structure (beta diversity) was implemented on the css-, tss-, and Hellinger-transformed data sets, wherein Bray-Curtis dissimilarity matrices were calculated and projected using principal component analysis (PCA) and principal coordinate analysis (PCoA) for data pattern exploration and community structure, respectively. Bray-Curtis dissimilarity matrices were further subjected to permutational multivariate analysis (PERMANOVA; 999 permutations), using the following model: ADONIS2 (Bray-Curtis_dist. ~ genotype, data=data, permutations = 999, strata=data$location), which examines the independent effects of plant genotype on the seed bacterial structure, while adjusting for the influence of sample location (as the strata parameter). Furthermore, the effect of host genotype on seed bacterial community was assessed for individual locations where at least five genotypes were collected. These locations include Jiangsu (number of genotypes, *n* = 51), Shanxi (*n* = 7), Heilongjiang (*n* = 13), Guangxi (*n* = 10), Beijing (*n* = 10), and Shaanxi (*N* = 8) (see Data S1 at https://doi.org/10.6084/m9.figshare.30823943). PERMANOVA was also individually implemented to examine the effect on the seed microbiome of other experimental factors as highlighted in the extensive metadata (Fig. 1; Data S1). Taxonomic community composition across tomato genotypes for the different locations was shown using stacked bar plots, representing the top 500 ASVs, using the GGPLOT2 package. Both alpha-diversity and beta-diversity analyses were implemented using RStudio and associated software packages (version 4.0.3).

The core microbiome, a concept that entails determining the consistent members of the microbiome, in space and time, is central in providing insights into potentially crucial members of the community, and in a broad sense refers to community members that are ubiquitous across samples (48). Therefore, different core tomato seed microbiomes were determined based on the prevalence thresholds of 50%, 70%, and 90% across samples. The core was represented using a trimmed phylogenetic tree to include only taxa with a relative abundance above 0.005% (*n* = 488), rendered using the Interactive Tree Of Life tool v6 (49) and annotated in R.

Random forest classification (i.e., set.seed(57938), ntrees=500, and cores=3), with unrestricted growth, was implemented on the tomato seeds microbiome data set, and the predictive microbial features for various tomato traits presented at genus taxonomic rank, using caret and randomForest package (v.4.6.14) in R with default parameters (50–52). The classifier was implemented on filtered data sets with a minimum average relative abundance threshold for ASVs of 0.05 across samples; wherein important features that were correlated with host genotype and location, as well as host traits including berry taste, shape, and color; resistance to insects (aphids and whiteflies) and TMV; yield, 1,000 seed weight in grams, and number of ovaries were determined. The selected top five features, ordered based on mean decrease in accuracy values, were presented. The Gini indices represent the contribution of individual ASVs to the model and provide information about the important microbial features. For each factor, 70% of the data set features were used for training, and 30% for testing, and the model accuracy was subsequently determined.

## RESULTS

### Seed bacterial diversity varies across genotypes and geographic locations of seed production

**Fig 2:**
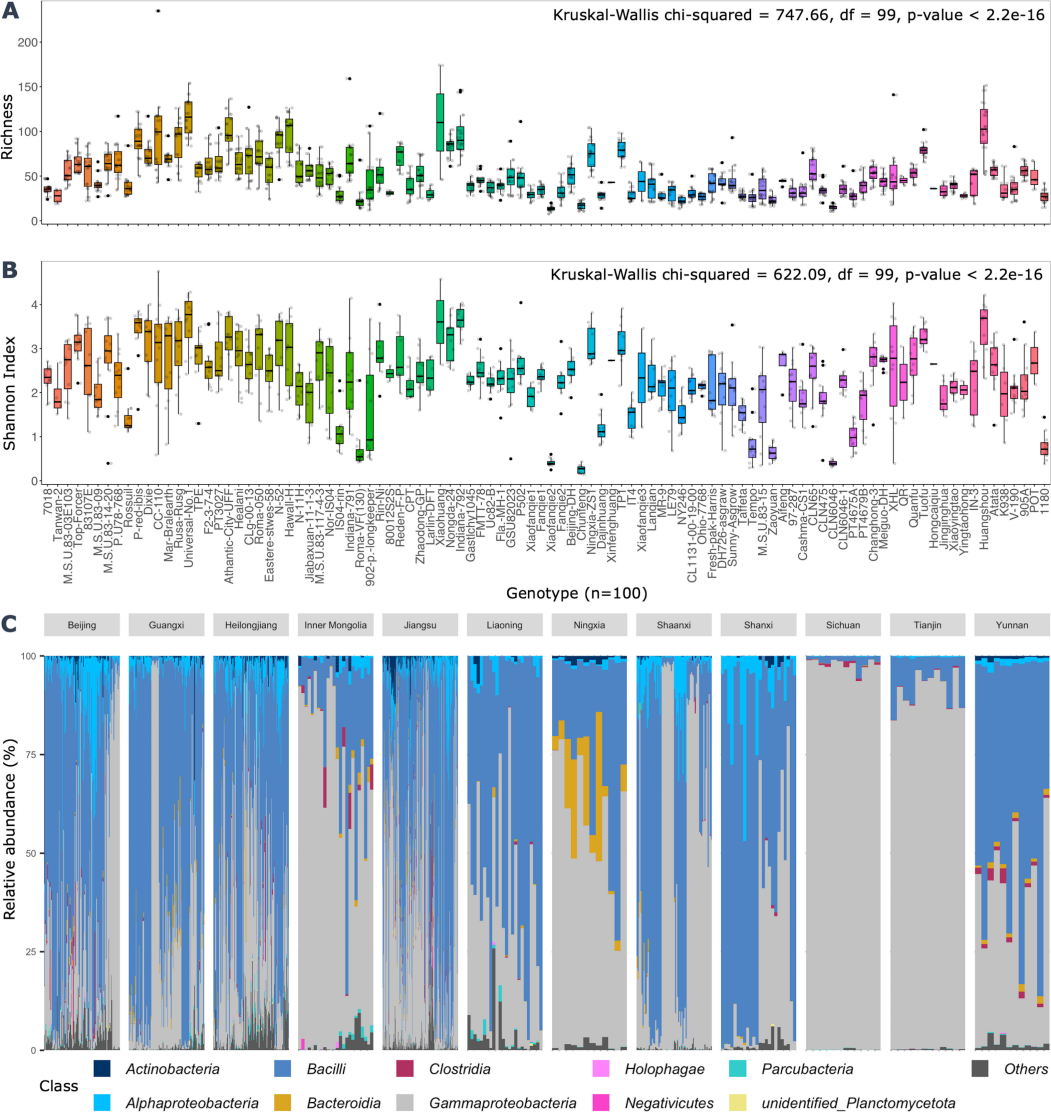
Seed bacterial diversity and composition. (**A and B**) Boxplots showing the seed bacterial diversity (i.e., richness and Shannon indices) across the tomato genotypes. Global significance differences between genotypes, as examined using Kruskal-Wallis tests, are shown in panels A and B. The boxplots represent sample distribution parameters (*n* = 12 samples per genotype) with the estimation of median, interquartile range, and the data points outside the boxplot area represent outliers. The color gradient represents different tomato genotypes (*n* = 100). Panel **C** shows tomato seed bacterial community composition of the top 500 ASVs across tomato genotypes, and grouped by the locations of seed production (*n* = 12). The stacked bar plot compositional representation is based on css-normalized data sets that rescale raw counts to ensure sample comparability while preserving library size variation.

The seed microbiome diversity was highly variable across tomato genotypes, with average observed ASV richness (51.3 ± 27.5, *n* = 1,039), median (46), and interquartile range (IQR = 34). The Shannon index (H′) parameters include mean (2.26 ± 0.89), median (2.31), and IQR (1.18) (Fig. 2A through 2B). Significant differences in alpha diversity were observed across genotypes (Kruskal-Wallis, *P* ≤ 2.2 × 10^−16^). The phylogenetic diversity and evenness (see Fig. S1B and D at https://doi.org/10.6084/m9.figshare.30823943), as well as the effective richness and Shannon diversity measures (Fig. S1E and F), showed a consistent pattern with the Shannon and richness indices, but with generally lower values ranging from 10 to 90 ASVs and 10–80 ASVs for effective richness and effective Shannon diversity, respectively. In contrast, the Simpson index and effective Simpson diversity showed a reverse trend to the abovementioned alpha diversity measures, wherein highly diverse samples had low Simpson index values (Fig. S1C and G). Regarding sample location (Fig. S2), significant differences in bacterial diversity were observed across the sampled locations (Kruskal-Wallis, *P* ≤ 2.2 × 10^−16^, df = 11); wherein samples obtained from Jiangsu were generally high in bacterial diversity, also representing a high portion of the analyzed tomato seed samples.

Analysis of the seed taxonomic composition revealed 1,181 genera in 109 different classes belonging to 38 phyla. Predominant phyla were Bacillota (53%), Pseudomonadota (43%), Actinomycetota (1.8%), and Bacteroidota (1.6%), respectively. By analyzing the top 500 ASVs, it became evident that the seed microbiome is dominated by four bacterial classes which collectively represented approximately 97.6% of the reads; they were comprised of Bacilli (51.7%), Gammaproteobacteria (37.5%), Alphaproteobacteria (5.2%), Actinomycetota (1.7%), and Bacteroidia (1.6%), respectively (Fig. 2C). The genus level taxonomic assessment showed a high abundance of *Lactobacillus* (30.7%), *Pseudomonas* (16.2%), *Leuconostoc* (7.2%), *Weissella* (6.6%), and *Pantoea* (4.5%); as well as *Acetobacter* (3.7%), *Ralstonia* (3%), *Lactococcus* (2.4%), *Kosakonia* (2.2%), and *Enterobacter* (2.2%), respectively.

### The host’s aphid and TMV resistance can be linked to high seed bacterial diversity

There were significant differences in bacterial diversity across the insect resistance continuum (Kruskal-Wallis, *P* ≤ 0.05 to 3.0 × 10^−5^, df = 5); tomato genotypes with high and general aphid resistance showed significantly higher Shannon indices, effective Shannon, and effective richness in comparison to their weak resistant counterparts (see Fig. S3B through D at https://doi.org/10.6084/m9.figshare.30823943). Meanwhile, no significant difference (*P* ≥ 0.05) in observed ASV richness was shown between general insect resistance (i.e., cotton bollworm and whiteflies) relative to the genotypes with weak or low resistance. This trend was reflected by the alpha diversity indices, like effective richness, Shannon index, effective diversity, phylogenetic diversity, Simpson index, effective Simpson diversity, and evenness (Fig. S3B through H, respectively). In addition, tomato genotypes that showed weak and general resistance to TMV had significantly higher observed ASV richness and phylogenetic diversity as compared to their highly resistant genotypes (Fig. S3A and E). However, alpha diversity measures, such as the effective richness diversity, Shannon index, effective Shannon diversity, Simpson index, effective Simpson diversity, and evenness, only showed significant differences between weak and highly resistant cultivars (Fig. S3B through D, then Fig. S3F through H, respectively).

### Specific tomato traits can influence the seed bacterial diversity

All host’s sensory and physical traits, except berry longitudinal and horizontal diameter, showed a significant influence on the tomato seed bacterial diversity. For instance, the number of ovaries was associated with the observed bacterial ASV richness, in which there were significant differences in alpha diversity indices across groups representing the number of ovaries (Kruskal-Wallis, *P* ≤ 1 × 10^−8^, df = 2) (see Fig. S5 at https://doi.org/10.6084/m9.figshare.30823943). Bacterial diversity also significantly varied across berry shapes (Kruskal-Wallis, *P* ≤ 0.005, df = 2), wherein globe-shaped tomato genotypes showed significantly higher bacterial alpha diversity as compared to plum- and oxheart-shaped tomatoes (Fig. S6). Furthermore, significant differences in bacterial alpha diversity were also observed for attributes, including berry color (Fig. S7), taste (Fig. S8), yield (Fig. S9), as well as seed weight (i.e., 1,000 seed weight in grams) (Fig. S10). Sweet tomatoes contained a significantly higher microbial diversity, richness, and evenness when compared to the sour-type tomatoes. The tomato berry dimensional attributes, such as longitudinal and horizontal diameter, showed no significant effect on the seed bacterial alpha diversity (Fig. S11 and S12). Moreover, the seed bacterial community was found to be completely covered within a sequencing depth ranging from 500 to 50,000 reads per sample (Fig. S13A and B). Details regarding the statistical analyses of the different alpha diversity indices, including richness, effective richness, Shannon index, effective Shannon diversity, phylogenetic diversity, Simpson index, effective Simpson diversity, and evenness, are shown in Data S4 to S11 at https://doi.org/10.6084/m9.figshare.30823943.

### The seed microbiome structure is mostly influenced by genotype and geographic location

**Fig 3:**
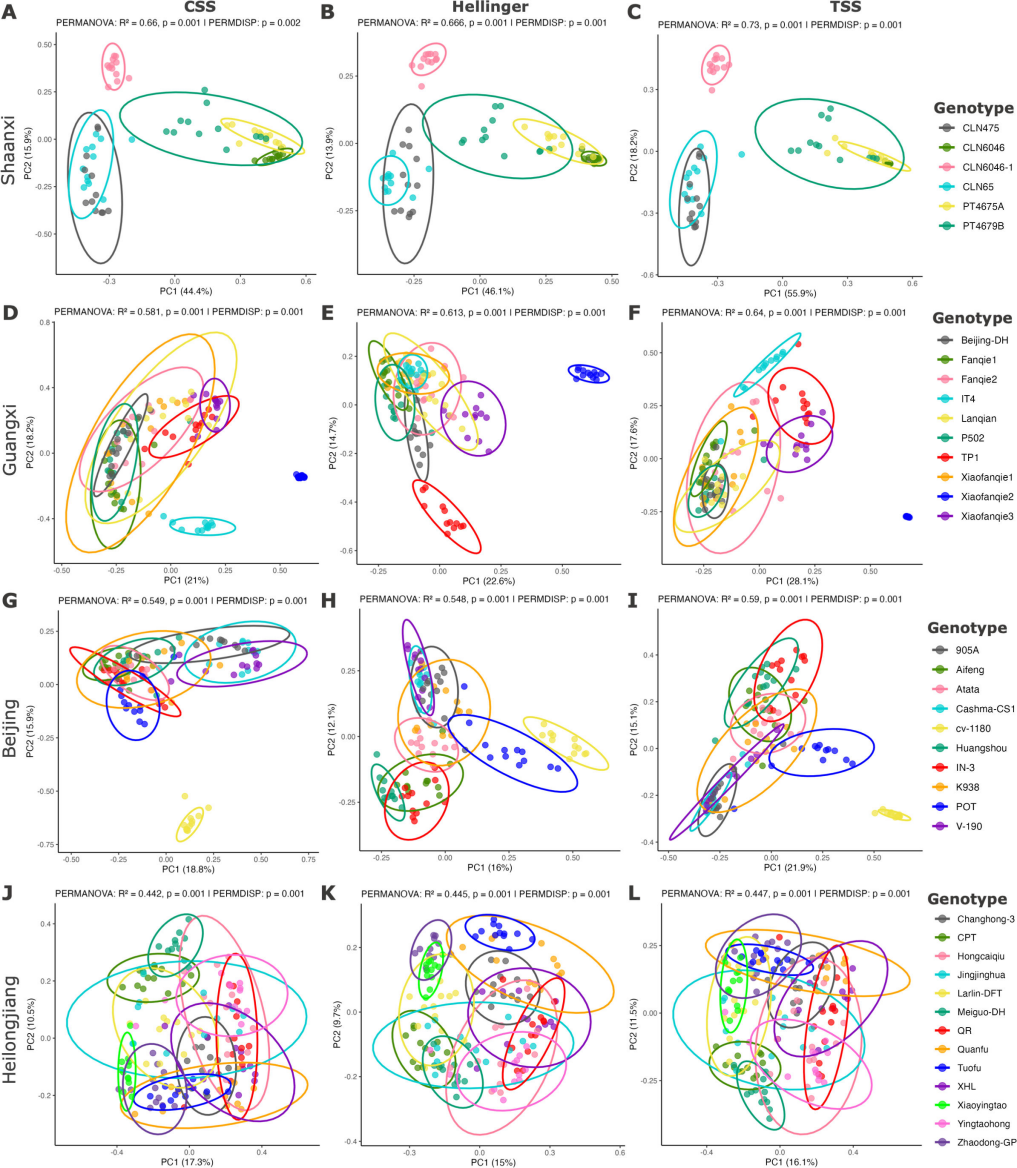
Location-specific variability in seed microbiome due to the effect of tomato genotype. (A**–C**) PCoAs showing the seed bacterial structure in Shaanxi for bacterial community data sets normalized using css, Hellinger, and tss, respectively; panels **D–F** represent the same for Guangxi, Beijing (**G–I**), and Heilongjiang (**J–L**). Colors represent the different tomato genotypes. The ellipse boundaries represent the 95% confidence of community clustering in the different samples.

To explore the influence of plant genotype and location on the seed microbiome, an ordination based on a Bray-Curtis dissimilarity matrix was conducted and visualized using PCA and PCoA to assess the sample distribution across genotypes (see Fig. S13C and D at https://doi.org/10.6084/m9.figshare.30823943) and locations (Fig. S13E and F). PERMANOVA showed the significant influence of tomato genotype (*P* = 0.001) on seed bacterial community variations and explained 56% of the variation in community structure; this was higher when compared to the effect of the parents’ locations (*R*^2^ = 11%, *P* = 0.001). Furthermore, the genotype effect was examined for individual host locations, including Shaanxi, Guangxi, Beijing, Heilongjiang, Jiangsu, and Shanxi (see Data S2 and S3 at https://doi.org/10.6084/m9.figshare.30823943); there was a significant influence of tomato genotype on the seed bacterial community with an effect size ranging between 20% and 73% (Fig. 3A through L; Fig. S14A through D). The variation in community structure was also represented by bacterial taxonomic composition, as represented for Jiangsu province (Fig. S14E).

### The influence of other host traits on tomato seed microbiome structure

**Fig 4:**
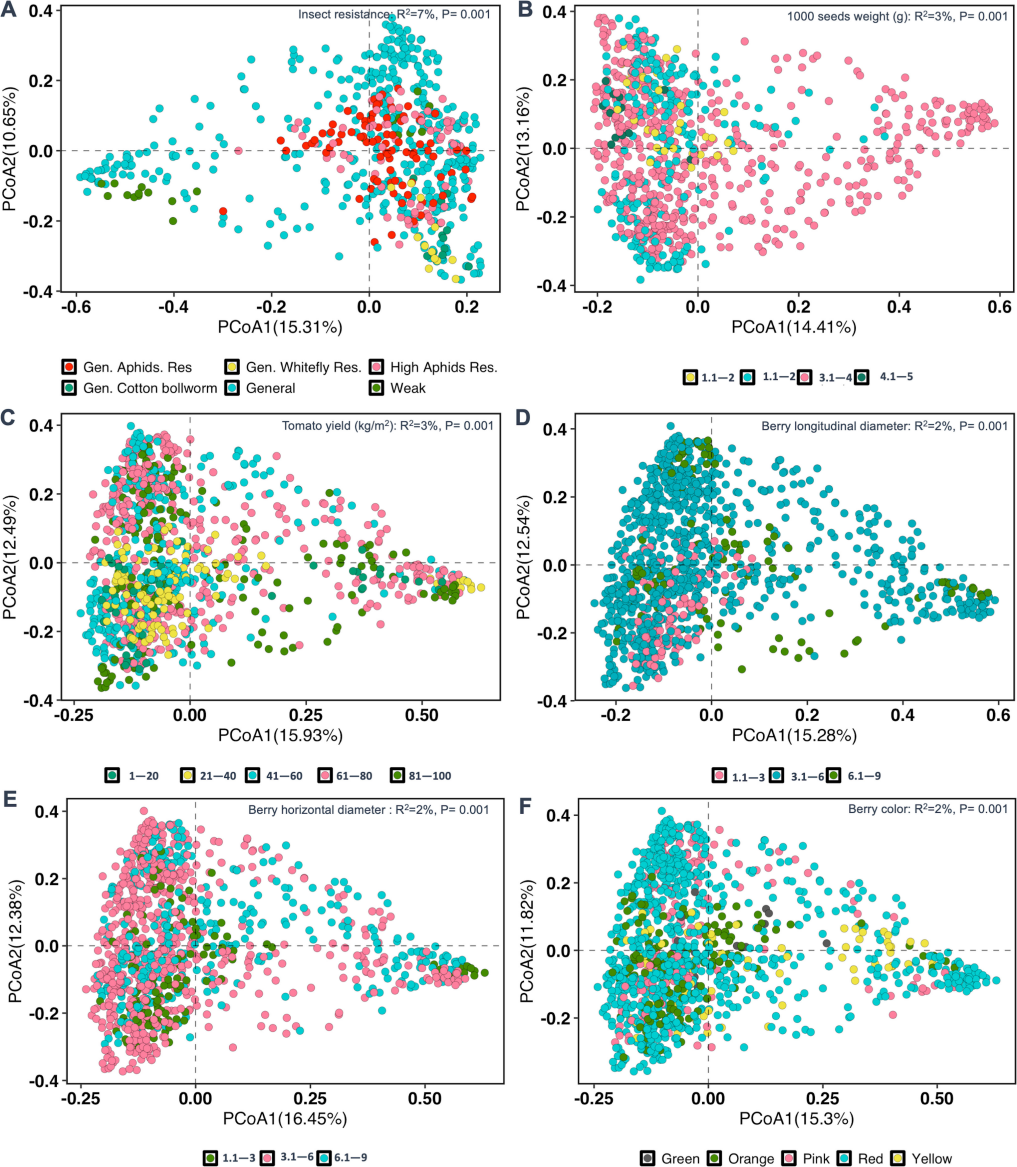
PCoA showing the seed microbial structure based on factors: insect resistance (**A**: *R*^2^ = 7%, df = 2, *P* = 0.001), 1,000 seed weight in grams (**B**: *R*^2^ = 3%, df = 3, *P* = 0.001), berry yield measured as weight in kg/m^2^ (**C**: *R*^2^ = 3%, df = 4, *P* = 0.001), berry longitudinal diameter (**D**: *R*^2^ = 2%, df = 2, *P* = 0.001), berry horizontal diameter (**E**: *R*^2^ = 2%, df = 2, *P* = 0.001), as well as berry color (**F**: *R*^2^ = 2%, df = 4, *P* = 0.001). The PCoAs are based on Bray-Curtis distance matrices on Hellinger-transformed microbiome data sets (using phyloseq-formatted microbiome data sets). Legends show the different categories of plant traits that were examined.

To explore the contribution of tomato sensory and physical traits on the seed bacterial community, ADONIS2 was performed using Bray-Curtis dissimilarity matrices of individual traits. We showed that tomato insect resistance significantly contributed to the seed bacterial variation (*R*^2^ = 7%, *P* = 0.001). A significant effect was also observed for the other traits, including the seed weight (*R*^2^ = 3%, *P* = 0.001), tomato yield (*R*^2^ = 3%, *P* = 0.001), berry longitudinal (*R*^2^ = 2%, *P* = 0.001) and horizontal diameter (*R*^2^ = 2%, *P* = 0.001), and berry color (*R*^2^ = 2%, *P* = 0.001), visualized by PCoA ordination in Fig. 4A through F, respectively. In addition, the number of ovaries (*R*^2^ = 2%, *P* = 0.001), berry color (*R*^2^ = 2%, *P* = 0.001), and berry taste (*R*^2^ = 2%, *P* = 0.001) had an effect. A significant, albeit lower influence of traits, like TMV resistance (*R*^2^ = 1.4%, *P* = 0.001), number of ovaries (*R*^2^ = 2%, *P* = 0.001), berry shape (*R*^2^ = 1.4%, *P* = 0.001), and berry taste (*R*^2^ = 0.4%, *P* = 0.001) was also revealed (see Fig. S15A through D at https://doi.org/10.6084/m9.figshare.30823943). The variation within seed bacterial community was further examined by computing the dispersion among the different experimental variables, where there were significant differences in dispersion (*P* = 2.2 × 10^−16^) across genotypes (df = 99) and location (df = 11) (Fig. S16A and B). Furthermore, significant dispersion was revealed across tomato traits, like insect resistance (df = 5, *P* = 2.4 × 10^−12^; Fig. S16C), seed weight (df = 3, *P* = 6.5 × 10^−8^; Fig. S16D), yield (df = 4, *P* = 8.6 × 10^−10^; Fig. S17A), longitudinal berry diameter (df = 2, *P* = 4 × 10^−6^; Fig. S17B), berry color (df = 4, *P* = 7.9 × 10^−9^; Fig. S17D), TMV resistance (df = 2, *P* = 4.0 × 10^−5^, ), number of ovaries (df = 2, *P* = 0.05; Fig. S17F), and berry shape (df = 2, *P* = 1.6 × 10^−8^; Fig. S17G). No significant differences (*P* ≥ 0.05) in dispersion were observed for traits, including berry diameter and taste (Fig. S17C and H, respectively). The variations in bacterial community, observed by PCoA ordination, were further reflected in bacterial taxonomic composition, where a predominance of the families *Lactobacillaceae*, *Pseudomonadaceae*, *Leuconostocaceae*, *Burkholderiaceae*, *Erwiniaceae*, *Acetobacteriaceae*, and *Streptococcaceae* was observed across the examined plant traits (Fig. S18). Interestingly, small-sized seeds were associated with a high number of taxa under the pseudo-group “others,” which represents low-abundant taxa (less than 0.1% relative abundance). Similarly, the status of tomato insect resistance (high vs low) was associated with high bacterial diversity, as well as a higher average abundance of bacterial taxa in the family *Lactobacillaceae*.

### A small core microbiome of 21 ASVs is shared across tomato genotypes

**Fig 5:**
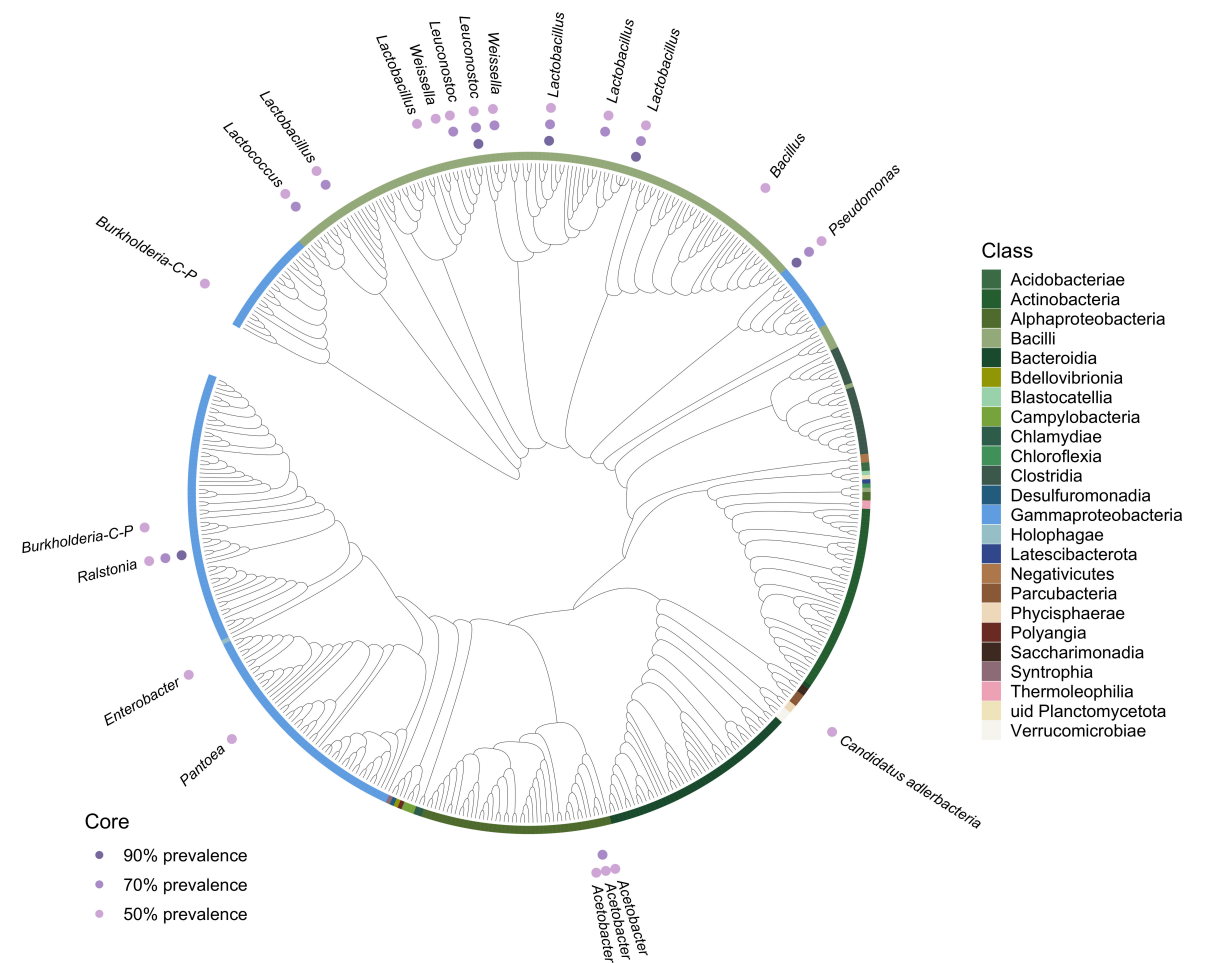
Taxonomy and prevalence of core microbiome across tomato cultivars. The phylogenetic tree shows 488 ASVs filtered using an abundance threshold of 0.005% and presented at class level. The color gradient (i.e., light to dark purple) shows variation in prevalence, in 50%, 70%, and 90% of the samples, respectively, for the classification of core members, which are described according to their genera.

We analyzed the prevalence of ASVs at different thresholds to identify bacterial taxa consistently associated with the tomato seed endosphere. A core microbiome of 21 ASVs was present in at least 50% of the samples. The observed core microbiome was further represented phylogenetically using a concise tree trimmed to represent 488 ASVs (Fig. 5). This set of bacteria was present across tomato cultivars and corresponded to the set of highly abundant microorganisms present in tomato seeds, with relative abundances above 0.1% up to 10% (see Fig. S19 at https://doi.org/10.6084/m9.figshare.30823943). Herein, members of Bacilli were predominant (*n* = 11), including multiple representatives of the genus *Lactobacillus* (*n* = 5). The second most prevalent class was Gammaproteobacteria (*n* = 6), followed by Alphaproteobacteria (*n* = 3) and one unidentified Planctomycetota. Other detected genera included *Acetobacter* (*n* = 3), *Burkholderia* (*n* = 2), *Weissella* (*n* = 2), *Leuconostoc* (*n* = 2), *Lactococcus* (*n* = 1), *Bacillus* (*n* = 1), *Pseudomonas* (*n* = 1), *Pantoea* (*n* = 1), *Enterobacter* (*n* = 1), *Ralstonia* (*n* = 1), and candidate *Alderbacteria* (*n* = 1), making up for a total of 12 different genera. At a 70% prevalence threshold, the core microbiome diversity at the genus level was largely maintained, with *Lactococcus*, *Lactobacillus*, *Weissella*, *Leuconostoc*, *Pseudomonas*, *Ralstonia*, and *Acetobacter* still detected. Notably, members of Bacilli remained dominant, reinforcing their potential ecological significance within the tomato seed microbiome. Only five ASVs were detected in more than 90% of the samples, highlighting a highly conserved subset of bacteria within the tomato seed endosphere. These included ASVs from the genera *Lactobacillus* (*n* = 2), *Leuconostoc* (*n* = 1), *Ralstonia*, and *Pseudomonas* (*n* = 2). These findings provide insight into the selective processes shaping the tomato seed microbiome and highlight candidate taxa for future functional studies.

### The seed microbiome as a lens for plant trait and environment prediction

**Fig 6:**
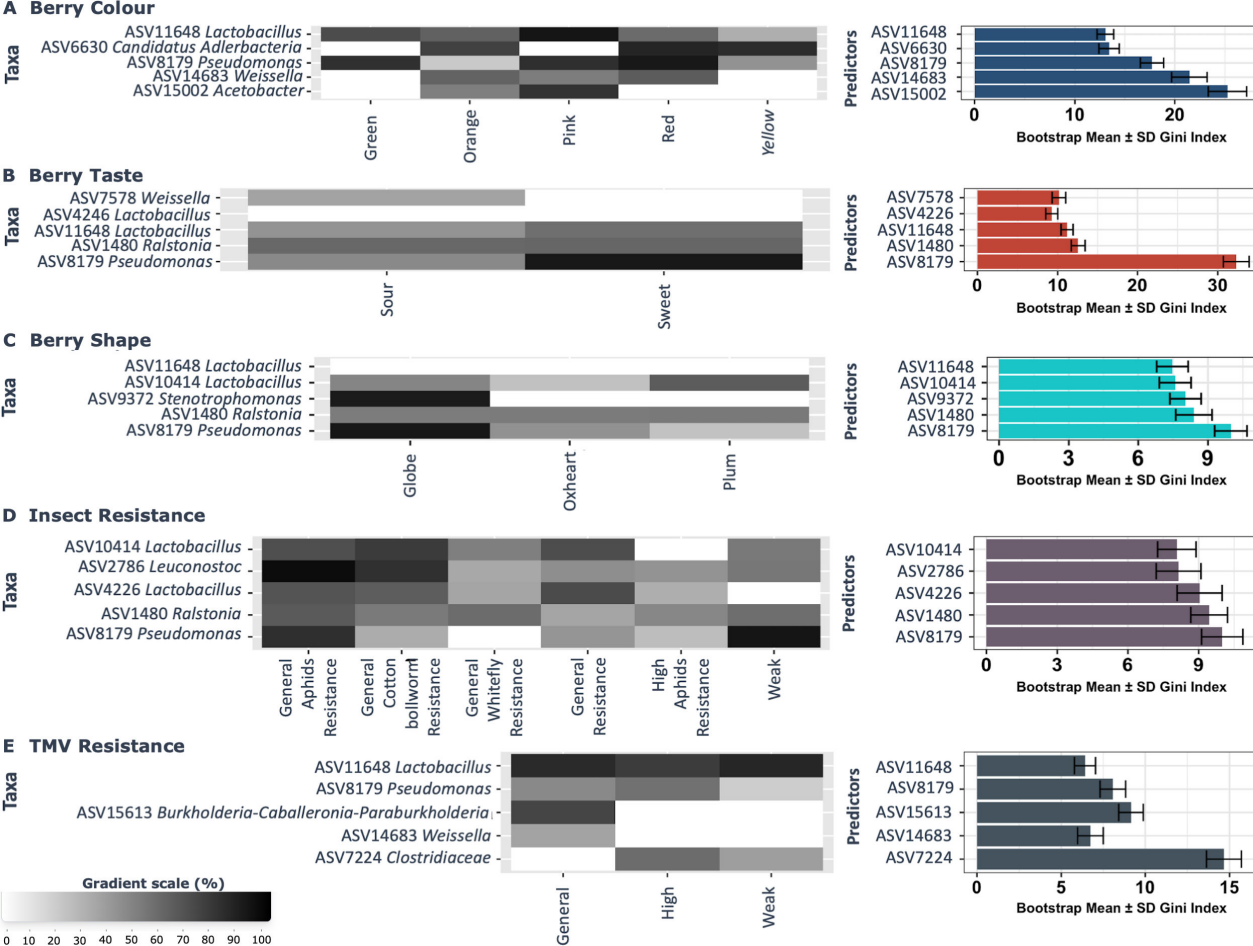
The seed microbiome can be used as a biomarker for host traits and the environment (location) where tomato plants were produced. (**A–E**) Heatmap-barplots showing the top five most important seed microbial features that can delimit the different host traits. The represented factors include berry color, taste, and shape, as well as insect and TMV resistance, respectively. The most important bacterial features are arranged in order of decreasing Gini index. The gradient color scale represents the percentage abundance of the taxa.

By implementing a random forest classifier on the data sets, we showed that the seed microbiome can delimit plant traits and parents’ locations with high accuracy, ranging from 0.77 to 0.90 (see Table S1https://doi.org/10.6084/m9.figshare.30823943), and that the majority of the key delimiting taxa also comprised the core seed microbiome. For instance, *Lactobacillus* (ASV10414) and *Ralstonia* (ASV1480) were shown to be the most important features in predicting sample location (Fig. 6A). *Acetobacter* and *Weissella* could best predict berry color (Fig. 6B). Meanwhile, *Pseudomonas* (ASV8179) was an important delimiter of tomato traits, such as berry taste and berry shape (Fig. 6C and D). The status of tomato insect resistance was associated with genera like *Pseudomonas* (ASV8179), *Ralstonia* (ASV1480), and *Lactobacillus* (ASV1480), while tomato TMV resistance could be distinguished by bacteria within the genus *Clostridiaceae* (Fig. 6E). Moreover, *Pseudomonas* (ASV8179) was also crucial in delimiting other host traits, such as tomato genotype, yield, seed weight, number of ovaries, and horizontal and longitudinal berry diameter (see Fig. S20A through F at https://doi.org/10.6084/m9.figshare.30823943). *Pantoea*, just like *Pseudomonas,* was important in distinguishing the different assigned ovary groupings. *Leuconostoc* and *Cupriavidus* were found to allow delimiting between the longitudinal berry diameter.

## DISCUSSION

Here, the so-far largest tomato seed microbiome data set was utilized to examine commonalities and differences across various host genotypes and to provide an accurate overview of the overall seed microbiome diversity and composition. Targeted analyses were aimed at elucidating the contribution of host genotype and location, along with other plant and production traits, to shaping the seed microbiome. We show that plant genotype is the main shaping factor of tomato seed microbiome diversity. This is in line with recent findings that indicated an overall effect of host plant genetics on the *Cannabis* seed microbiome of approximately 53% (17), and also comparable to more general findings quantifying host genetics effects on the seed microbiome, which were in a range from 22% to 30% (16). The influence of the plant genotype on the seed microbiome was found to not only be substantial in agroecosystems (53, 54) but also in natural environments (55). Geographic location, as shown in the current study, also contributed significantly to variations in seed microbiomes. Previously, it was shown to be an integral contributor to plant microbiome assembly (13, 56). Overall, our findings are supported by prior research citing a higher influence of plant genetics than geography in influencing the plant microbiome (57).

Tomato is a desired host for aphids that spread viral infections, like TMV (58, 59). Our data indicate that tomato genotypes with aphid resistance were associated with a high bacterial diversity and evenness in seeds relative to their non-resistant counterparts. Plant host immunity is genotype dependent and essential in mediating plant defense against phytopathogenic infections caused by insect pests and viruses (60–62). Seeds were previously shown to serve as shuttles of beneficial microorganisms that are essential for plant health. This knowledge can be harnessed for the targeted engineering of microbiomes through the introduction of selected microbes that may enhance plant resilience (63, 64), with potential legacy effects of the applied microbes in the next plant generation (22, 23, 63).

The tomato seed microbiome was shown to be specialized, with few members ubiquitous across genotypes, constituting the core microbiome. The observed plasticity in bacterial diversity across genotypes reinforces findings of a seed microbiome meta-study, in which a similar diversity range was observed for the tomato seed microbiome by combining several comparatively small studies (16). We presume that a specialized and consistent seed microbiome could be a product of plant-microbiome coevolution, wherein plants select part of their lifetime-assembled microbiome as inoculants for re-establishing the next plant generation. A few members of the core seed microbiome, like *Pseudomonas*, *Pantoea*, *Ralstonia*, and *Lactobacillus*, were shown to predict and delimit various plant traits. Recently, integrated canopy imaging of emerging potato shoots and potato tuber microbiome fingerprints was used in the prediction of potato tuber vigor (13). As remarked by the authors (13), and as observed in the current study, it is unclear whether the important microbial features that are observed to delimit various plant traits are a cause or consequence of the very traits they help to predict.

Across genotypes, tomato seed-associated bacteria were mainly assigned to the phylogenetic groups represented by Bacillota, Pseudomonadota, Actinobacteriota, and Bacteroidota; as previously described (16, 65). High proportions of these phyla can be explained, in part, by their prevalence in ecosystems that plants and seeds encounter in the surrounding soil (56, 65, 66). The tomato seed microbiome was dominated by taxa comprised of *Pseudomonas*, *Lactobacillus*, *Leuconostoc*, *Lactococcus*, *Pantoea*, *Ralstonia*, and *Acetobacter*, many of which are ubiquitous members of the seed core microbiome as shown in previous research (16). We also observed a high abundance of bacteria within the family *Lactobacillaceae,* which could be potentially attributed to their wide association with pollinating insects, with the consequence of being integrated into seeds (67, 68). Resistant tomato cultivars were associated with a high bacterial diversity, together with the presence of potential disease-protective bacterial genera, such as *Pantoea* and *Pseudomonas* (69–72). *Pantoea* has been recently shown to be consistently vertically transmitted across wheat generations via seeds (23). This and its frequent occurrence in seeds of different plant species provide indications of co-evolved mechanisms that enable a continuum of reciprocal interactions between hosts and microbes.

### Conclusions

We found that the tomato genotype is the main contributor to seed microbiome variation. The genotype-dependent seed microbiota is also reflected in various plant traits, such as the resistance of tomatoes to insect pests. We also found that the geographical location where the parent plant was grown plays an essential role through the provision of a bulk source of different microorganisms from which plants can assemble the genotype-dependent and specialized seed microbiome. Given the central role of seeds in ensuring plant generational continuity and the transmission of seed microbiomes (15, 23, 73), understanding the factors that shape the seed microbiota is essential for redefining crop improvement programs to optimize seed microbiomes (16). Moreover, there is increasing evidence regarding the role of specific plant microbiome-regulating genes, so-called *M* genes, which are central for the selection of plant-beneficial microbes and therefore could be key targets for sustainable crop breeding frameworks (69, 74–76).

## Data Availability

Raw sequence data (from 16S rRNA gene fragment sequencing) reported in this work were deposited into the European Nucleotide Archive (ENA) under project accession number PRJEB84389. All custom scripts for use in the analysis of the data can be found on GitHub (https://github.com/kaboyo/Tomato-seed-microbiome).
